# Computational Studies on T2Rs Agonist-Based Anti–COVID-19 Drug Design

**DOI:** 10.3389/fmolb.2021.637124

**Published:** 2021-08-17

**Authors:** Premnath Dhanaraj, Indiraleka Muthiah, Mahtabin Rodela Rozbu, Samiha Nuzhat, Mosae Selvakumar Paulraj

**Affiliations:** ^1^ Department of Biotechnology, School of Agriculture and Biosciences, Karunya Institute of Technology and Science (Deemed to be University), Coimbatore, India; ^2^ Department of Biotechnology, Mepco Schlenk Engineering College, Sivakasi, India; ^3^ Department of Science and Math Program, Asian University for Women, Chittagong, Bangladesh

**Keywords:** coronavirus (COVID−19), molecular docking, T2Rs agonist, drug design, spike (S) glycoprotein

## Abstract

The expeditious and world pandemic viral disease of new coronavirus (SARS-CoV-2) has formed a prompt urgency to discover auspicious target-based ligand for the treatment of COVID-19. Symptoms of novel coronavirus disease (COVID-19) typically include dry cough, fever, and shortness of breath. Recent studies on many COVID-19 patients in Italy and the United Kingdom found increasing anosmia and ageusia among the COVID-19-infected patients. SARS-CoV-2 possibly infects neurons in the nasal passage and disrupts the senses of smell and taste, like other coronaviruses, such as SARS-CoV and MERS-CoV that could target the central nervous system. Developing a drug based on the T2Rs might be of better understanding and worth finding better molecules to act against COVID-19. In this research, we have taken a taste receptor agonist molecule to find a better core molecule that may act as the best resource to design a drug or corresponding derivatives. Based on the computational docking studies, the antibiotic tobramycin showed the best interaction against 6LU7 COVID-19 main protease. Aromatic carbonyl functional groups of the molecule established intermolecular hydrogen bonding interaction with GLN189 amino acid and it showed the two strongest carbonyl interactions with receptor protein resulting in a glide score of −11.159. To conclude, depending on the molecular recognition of the GPCR proteins, the agonist molecule can be recognized to represent the cell secondary mechanism; thus, it provides enough confidence to design a suitable molecule based on the tobramycin drug.

## Introduction

A new strain of single-stranded RNA virus, belonging to the Coronoviridae family, brought the world to a halt, presenting 2020 with the coronavirus pandemic. HCoV-229E, HCoV-OC43, SARS-CoV or SARS-CoV-1, SARS-CoV-2, MERS-CoV (Middle East respiratory syndrome coronavirus), HCoV-NL63, and HCoV-HKU1 are seven members known (to date) to be part of the coronavirus family (Nichols et al., 2008; Song et al., 2019; Su S et al., 2016; Kuek and Lee, 2020). The entry receptors present in this virus vary across the members. For example, HCoV-229E utilizes human aminopeptidase N (Buzon et al., 2011; Wang et al., 2000; HCoV-OC43 utilizes either human leukocyte antigen (HLA) class I or sialic acids (Collins, 1993; Owczarek et al., 2018), SARS-CoV and SARS-CoV-2 utilize angiotensin-converting enzyme 2 (ACE2; Song Z et al., 2019; Sungnak W et al., 2020), and MERS-CoV utilizes dipeptidyl peptidase 4 (Nichols et al., 2008; Su et al., 2016; Owczarek et al., 2018; Song et al., 2019). Among the seven members, HCoV-229E and HCoV-OC43 have been widely studied, known to cause mild flu-like symptoms, unlike the pathogenic SARS-CoV, SARS-CoV-2, and MERS-CoV strains that can induce serious respiratory distress and pneumonia and be fatal (Su S et al., 2016; Song Z et al., 2019; Mason, 2020 ). In most of the patients affected with the virus of Coronaviridae family, the sensory neurons in their nasal passage are affected, resulting in loss of smell and taste that demonstrates to be a hallmark symptom for COVID-19. Other common symptoms of COVID-19 infection include respiratory dysfunctions, fever, aches, tiredness, and coughing so on (Ul Qamar et al., 2020).

However, studies have already demonstrated that bitter taste receptors have a significant correlation with COVID-19 infection (Margulis et al., 2020). Barham et al. (2020) argue that SARS-CoV-2 is the most responsible strain of Coronaviridae that spiked the global pandemic of 2019–2020. This virus is highly pathogenic for which, despite the urgency, a prompt, safe, and effective drug development against this virus has become quite difficult (El Zowalaty and Järhult, 2020; Zu et al., 2020). Now, novel approaches for the design and discovery of drugs are being utilized to find efficient therapeutic drug candidates for COVID-19. Molecular docking is a promising tool for drug discovery and development that studies the interaction of ligand (drug) molecules inside the binding pocket of a target protein (receptor) (Narkhede et al., 2020; Yu et al., 2020). Molecular docking, a structure-based drug design approach, can help to identify the essential amino acid interactions between the selected protein and generated ligands with low energy conformation (Carlesso et al., 2019; Shah et al., 2020). Shah et al. (2020) has observed significant docking potentials in between bitter taste receptors and SARS-CoV-2. Specific transduction mechanisms are usually involved with corresponding senses of different types of tastes. In the case of bitter taste receptors expressing in the presence of type 2 family genes of different taste receptors, G-protein–coupled receptors (GPCR) bind with water-soluble molecules (Andres-Barquin and Conte, 2004). The bitter taste receptors, known as T2Rs, assure innate immunity mostly for ciliated sinonasal epithelial cells present in the first layer of upper airway immunity that interacts with SARS-CoV-2 causing the breakdown of immune responses (Maina et al., 2018; Aoe, 2020). Here, GPCR works as the primary receptor of bitter taste receptors (T2Rs). Spontaneous molecular docking potential in between these two complexes works as an influencing factor in this regard. In this regard, the concept of agonistic and antagonistic molecules is relevant. Here, the potential drug should agonist with the receptor protein to assure suitable ligand binding.

Unfortunately, drug designing and drug development initiatives to fight against COVID-19 involves several challenges. Despite the urgency of effective drug designing, the processes involved in this regard are quite lengthy and complicated. Frequent mutation in the viral genome is one of the major challenges in such drug designing. The pace at which the SARS-CoV-2 is mutating cannot be made sense by concurrent scientific understanding (Callaway, 2020). Such mutations often cause inefficacy of the universally designed drugs for COVID-19. Unfortunately, this virus is mutating to adopt a more contagious form making it more widespread compared to the initially detected types of the virus (University of Texas at Austin, 2020), which requires speedier innovations of drugs to stop the spread of the virus. Additionally, the scientific procedure for the market launching of a drug is quite lengthy and complicated for which even if several laboratories have predicted potential antiviral drugs for COVID-19, its validity testing and approval require a good amount of time. Besides, since the potentially designed drugs for COVID-19 might have several different side effects, the clinical trials of these drugs are permitted limitedly to minimize unprecedented adverse effects (Shi et al., 2020). Thus, drug designing and drug development procedure associated with this certain virus is yet a complex task.

However, considering the corresponding drug development-related challenges and association of bitter taste receptors with SARS-CoV-2, this study aims to predict a suitable structure for a drug molecule that may have suitable molecular recognition with GPCR proteins.

## Materials and Methods

**FIGURE 1:**
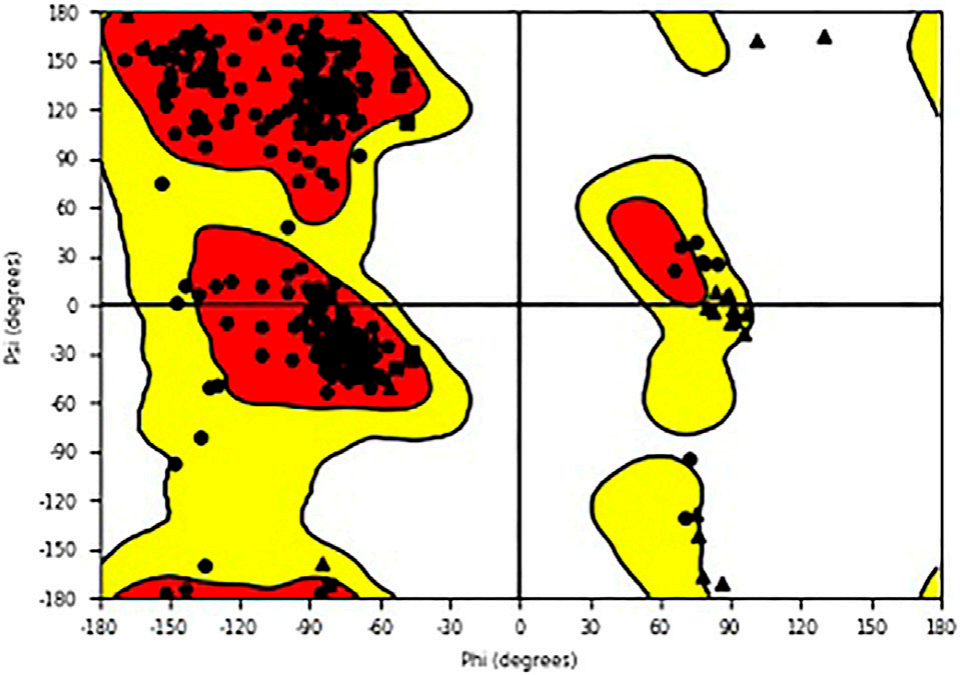
6LU7 optimized protein structure using Ramachandran plot.

The study used Schrödinger docking software to seek core molecules that can efficiently inhibit the pathway of COVID-19 pathogen as it invades cellular membranes, spreading within the body. For T2R, a mechanism that was experimentally validated was used (Liu, K et al., 2018). The docking interaction of T2R agonist along with 6LU7 (COVID-19 main protease in complex with an inhibitor N3, chain A) served as docking control for this study. The 6LU7 viral protein structure was taken from the PDB database. The quality of the model was checked using the Ramachandran plot for residues in favorable and allowed regions (Figure 1). The grid area around the extracellular site of the receptor was generated (x = 37A, y = 62A, and z = 59A) for ligand docking. The 2-dimensional (2D) chemical structures of tobramycin, azithromycin, structure-2D (3,371), C4-HSL, NHQ (2-N-3HQ), 3-oxo-C12-HSL, cromolyn, diphenhydramine, levofloxacin, C8-HSL, and quinine were obtained from the PubChem database, with their 3D structures optimized using the LigPrep module in Schrodinger. During the ligand optimization process using molecular mechanics, biochemical properties of the selective ligands were optimized. The particular drugs were chosen based on their bitterness score performed by previous studies on T2R using the E-tongue analysis and other pharmacological characterization (Jaggupilli et al, 2019). The ligands were docked onto spike protein structure using the Glide module, SP, and XP. Stable structural molecules were determined by observing the glide score produced by the computational docking, and the best poses were selected. Glide scores with greater negative values were significantly considered that suggested the potential structure. The research observed the molecular dynamics and interaction of T2Rs agonists drug molecules (Premnath et al., 2015) with surface spike proteins in COVID-19. The simulated complex of ligand and spike proteins was analyzed to study the biomolecular interactions with chemical bondings such as hydrogen bonding and hydrophobic bonding and the binding affinity of the receptor. Schrodinger’s PyMOL visualizer was chosen to generate quality images (Muthiah I et al.,2020).

## Results and Discussion

**FIGURE 2:**
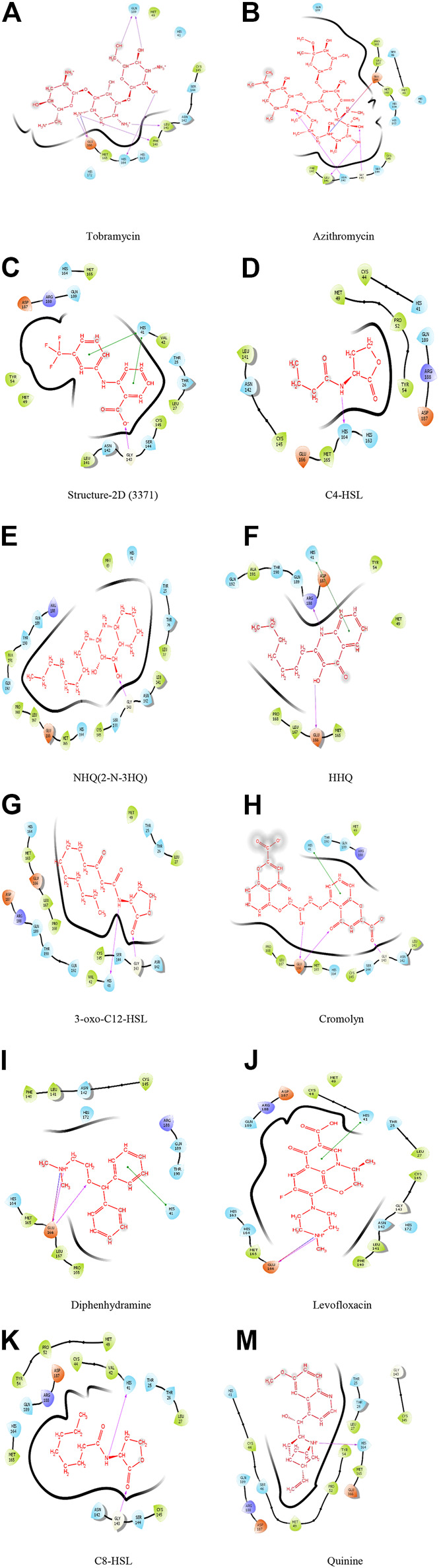
Molecular interaction of ligands with 6LU7.

**FIGURE 3:**
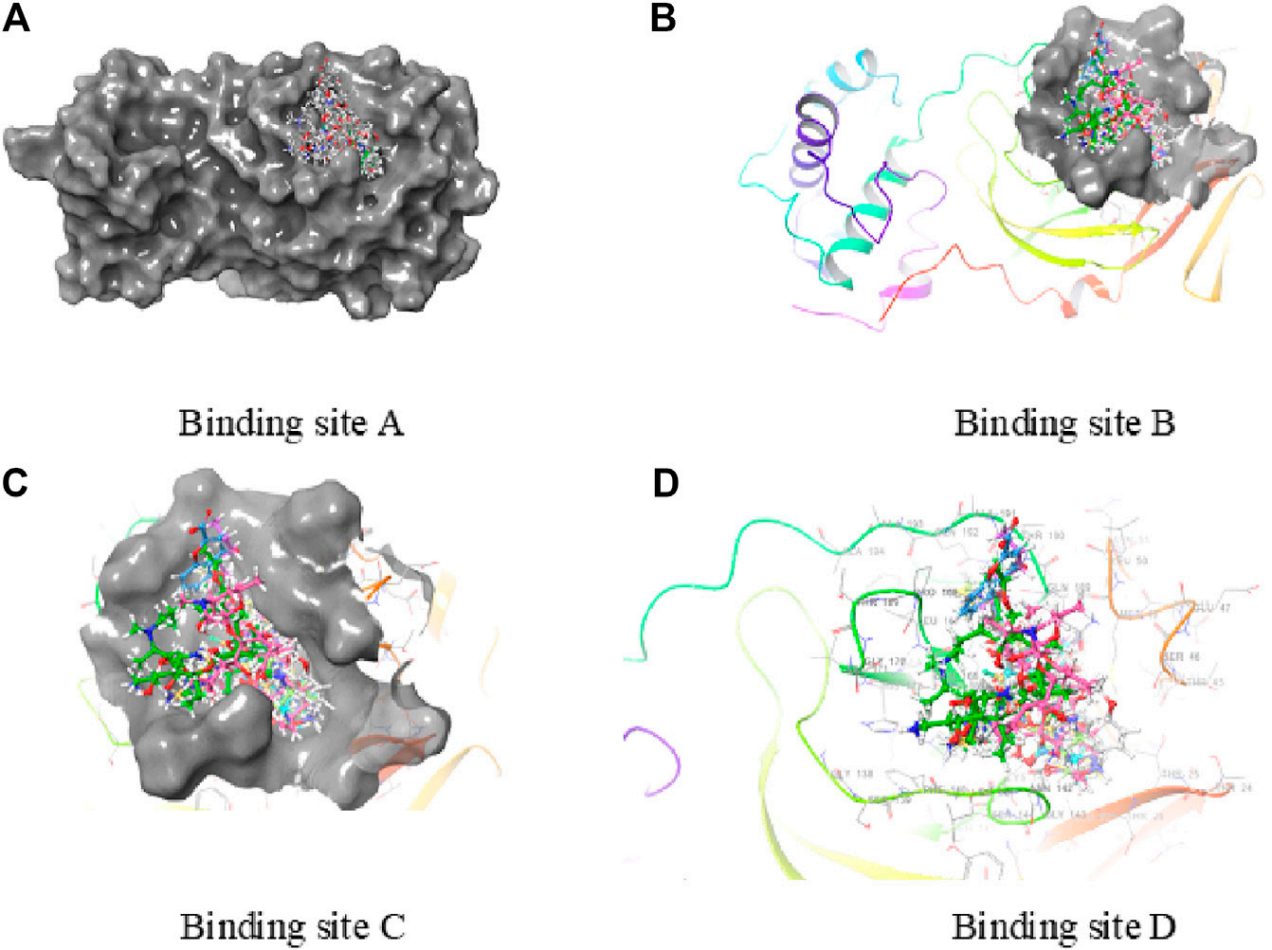
Active binding sites of 6LU7.

Neutrophils, monocytes, and lymphocytes can express bitter taste receptors (T2Rs), being involved in immune response (Maurer et al., 2015; Gaida et al., 2016; Sharma et al., 2017; Jaggupilli et al., 2019), and was observed to show decreased features of asthma in mural studies (Sharma et al., 2017; Jaggupilli et al., 2019). Studies have shown the particular antibiotic treatment to reduce inflammation by activating T2Rs, for certain diseases affecting airways (Jaggupilli et al., 2019). This research, therefore, studied the docking traits for theT2Rs agonist drugs/compounds of tobramycin, azithromycin, structure-2D (3,371), C4-HSL, NHQ (2-N-3HQ), HHQ, 3-oxo-C12-HSL, cromolyn, diphenhydramine, levofloxacin, C8-HSL, and quinine in order to facilitate a suggestive molecular structure to inhibit the rapid spread of COVID-19 pathogen. Successful docking of all the ligands revealed significant binding with the target protease. The results revealed strong interactions between the potential drug candidates against the T2R agonists, where tobramycin had the highest glide score of −11.159, followed by azithromycin and structure-2D (3,371). The drug quinine was observed to have the least docking score of −4.04 (Table 1), denoting the weakest docking potential. Here, Table 1 illustrates the docking molecules along with their corresponding structures for intermolecular docking, docking score, and glide score. Understanding the docking score and glide score is necessary to assure rapid, accurate, and sophisticated docking potential measurements with binding moiety (Figure 2, Figure 3) (Friesner et al., 2004).

**TABLE 1:** Molecular interaction characterization showing the docking score of T2Rs agonist onto the COVID-19 virus spike protein structure.

S. no	Docking molecule	Glide score (kcal/mol)	Glide energy (kcal/mol)	No. of H bonds	Interacting residues
1	Tobramycin	−11.159	−11.298	6	GLU 166, HIS 164, PHE 140, LEU 141, GLN 189
2	Azithromycin	−6.159	−6.172	3	LEU 141, ASN 142, GLY 143, GLU 166
3	Structure-2D (3,371)	−6.146	−6.147	1	GLY 143, HIS 41
4	C4-HSL	−5.328	−5.328	1	HIS 164
5	NHQ (2-N-3HQ)	−5.259	−5.26	1	GLY 143
6	HHQ	−4.918	−4.918	2	GLU 166, ARG 188, HIS 41
7	3-Oxo-C12-HSL	−4.895	−4.934	2	GLY 143, HIS 41
8	Cromolyn	−4.78	−4.78	3	GLU 166, GLY 143, HIS 41
9	Diphenhydramine	−4.682	−4.696	2	GLU 166, HIS 41
10	Levofloxacin	−4.491	−5.712	1	GLU 166, HIS 41
11	C8-HSL	−4.157	−4.157	2	GLY 143, HIS 41
12	Quinine	−4.04	−4.054	1	HIS164

Analysis revealed that among all the different molecules, tobramycin has strong interactions between the aromatic carbonyl functional groups and GLN189 amino acid. Two strong carbonyl interactions with receptor protein had a glide score of −11.159, making the drug tobramycin a potential model to design drug molecules or create drug derivatives. The drug azithromycin demonstrated two side chain bonding (between aromatic carbonyl groups and LEU141 and GLY143), along with the presence of a hydrogen bond (GLU166 and carbonyl group) (Figure 2). The docking result hence suggested that the binding energy from the docking of 6LU7 with the ligands tobramycin and azithromycin was significant compared to the other ligand molecules, with a docking score of −6.159 KJ/mol and −6.146 KJ/mol, respectively (Table 1). The docking results, thus, suggested that the drug molecule tobramycin had a greater capability to inhibit SARS-CoV-2 since it demonstrated high-affinity interaction with the T2R agonist molecule, similar to the docking interaction between T2R and 6LU7 COVID-19 protease. The results also indicated the presence of six side-chain bondings for tobramycin (Table 1), where the intermolecular hydrogen bonding was observed between amino acid GLN189 and aromatic carbonyl groups, alongside other strong carbonyl interactions. Such intermolecular bonding enables the drug–ligand interaction to be particularly stable, relative to the other docked complexes. This is how in the case of tobramycin a stronger interaction is caused due to a higher number of hydrogen bonds which is not alike for other molecules. For instance, the molecule NHQ (2-N-3HQ) consists of one hydrogen bonding interaction as side chain bonding between GLY143 and the aromatic carbonyl groups (Figure 2). The major interaction of the drug quinine was characterized to have one side chain interaction with HIS164 and hence had the least potent docking and glide score (Table 1).

Other drugs and molecular complexes tested, such as diphenhydramine, levofloxacin, HHQ, 3-oxo-C12-HSL, cromolyn, and C8-HSL, respectively, also contained side chain, backbone, and hydrogen bonding interactions. However, the total number of bonding interactions was relatively less than that in tobramycin. Although the drugs had the same binding pocket with different secondary interactions depending on their orientation, the drug tobramycin is speculated to be promising to design a molecule based on its structure. Azithromycin, levofloxacin, C4-HSL, and structure-2D also showed prominent binding interaction with T2R agonist, which accounted to study the number of hit molecules, perform virtual screening, and optimize lead compounds. Studying the docking properties and drug’s molecular dynamics, based on the interactions of the taste receptor and spike protein, upon modifying the complex (the drug–ligand interaction) the pathway where COVID-19 invades adjacent host cells, via cytoplasmic membranes, can be altered and prevent the spread of the virus.

## Conclusion

The docking results yielded various interactions with the T2Rs agonist molecules, where some ligands were more favorable than others. The drug, tobramycin, had the highest affinity for T2Rs, followed by azithromycin. Depending on the molecular recognition, the GPCR proteins are highly recognized as the agonist molecule to represent the cell secondary mechanism to give confidence in designing a new derivative of the tobramycin drug, to biochemically inhibit the prominent spread of COVID-19.

## Data Availability

The raw data supporting the conclusions of this article will be made available by the authors, without undue reservation.
