# Supplementary material for: Computational Studies on T2Rs Agonist-Based Anti–COVID-19 Drug Design
Source: Front Mol Biosci. 2021 Aug 17;8:637124. doi: 10.3389/fmolb.2021.637124 (PMC8416165; doi:10.3389/fmolb.2021.637124)
Supplement: Supplementary file 1 [file Presentation1.pdf]

Molecular Docking studies (Experiment protocol):

Glide module of Schrodinger was used to carried out Molecular docking interaction studies in which, through active site (based on swissprot mutagenesis active site reference) based docking approach with Methyl derivatives.

Set of ligand structures with receptor grid has used for flexible docking based on Monte Carlo based simulated algorithm in Glide v5.7 in which Glide standard precision (SP) and extra precision (XP) were performed. Then 10–30% of final ligand poses was selected for subsequent re-docking using XP. Ligand poses, which have high score in SP docking interaction analysis, were further used in XP docking. XP mode is more accurate than SP mode because it can screen out the false positive. XP docking was more efficient and accurate as its run time is longer than standard precision docking (SP). In grid-based docking technique, the receptor is basically rigid. XP was developed to place the ligands that bind to the receptor in particular conformation. The best posse of each ligand was ranked based on Glide XPG. In each XP docking more than 50 ligand poses were generated which pass through initial screening are further subjected to evaluation and minimization of grid approximation. Scoring is then carried on energy minimized poses of ligand-protein complex to generate Glide score.

MD simulations were performed for the complex of Bitter taste series of agonist and antagonist derivatives with protein complex using Desmond 3.2 software, incorporating OPLS-2005 force field for 20 ns (nanosecond) simulation time. The Bitter taste series of agonist and antagonist complex with periodic boundary size of 100 × 100 × 100 Å was solvated in a orthorhombic box with conditions by adding TIP3P water molecules. To balance the net charge of the system the whole system was neutralized by adding counter ions Na^+ and Cl^-.In

Desmond, using default protocol which made up of a series of restrained minimizations and MD simulations where the whole system equilibration was carried out. Protein molecules and its initial coordinates were slowly relaxed without deviation during simulation. For restraining the non-hydrogen solute atom for 200ns simulation time the minimized system was relaxed with NPT (number of atom, pressure, and temperature) ensemble. Methyl derivatives complex MD simulation system was composed of 21,248 atoms. The whole system pressure and temperature was maintained at1.01325 bar and 300 K respectively. Particle-mesh Ewald method was used to compute the long-range electrostatic interactions and van der waals (VDW) cut-off was set to 9 Å.To analyze the simulated stability of the Bitter taste series of agonist and antagonist complex hydrogen bond geometry constraints of the full system were satisfied using SHAKE algorithm during simulation. The RMSD and energy fluctuation were calculated by analyzing the dynamic behavior and structural changes of the complex. The root mean square fluctuations (RMSF) for the backbone and side chain of each amino acid residue of main proteinase (6LU7) were analyzed. Further Bitter taste series of agonist and antagonist complex was analyzed and monitored for the stability in hydrogen bond interactions.

Molecular dynamics simulation studies:

MD simulation of Bitter taste molecule of Tobramycin - main proteinase (6LU7) complex. (A) MD simulation time Vs. RMSD of the backbone atom of Tobramycin - main proteinase (6LU7) (B) RMSD of heavy atom (ligand) of Tobramycin - main proteinase (6LU7)

RMSD of backbone atoms was observed between 0-1.8 Å, whereas for Tobramycin 0-2 Å and compound 2 1-3 Å value was observed. The root mean square fluctuation (RMSF) of were calculated by averaging over all atoms of given

residues were showed in trajectory. RMSF of most of the residues were within the limit of 2.5 Å, but root mean square fluctuation for few residues exceeds 3.0 Å. Small conformation changes were observed due to the lower atomic fluctuation in active site residues and its back bone atoms. Energy plot, RMSD plot and RMSF plot reveals the Tobramycin -protein docked complex were stable throughout the MD simulation. Tobramycin with main proteinase molecular interactions were monitored to assess the structural flexibility of the docked complex. During MD simulation, the hydrogen bonding patterns of Tobramycin with main proteinase (6LU7) docked complex were reproduced-bonds formed between Tobramycin with main proteinase (6LU7) complex during 20,000 ps MD simulation. Interaction of Tobramycin with main proteinase (6LU7) Protein active site residues involved in hydrogen bonding was monitored After MD simulation.

RMSD Plot for 6LU7-TOB

Root mean square deviation (RMSD) computes the average distance between the backbone atoms of starting structure (reference structure) with simulated structures (frame by frame) when superimposed

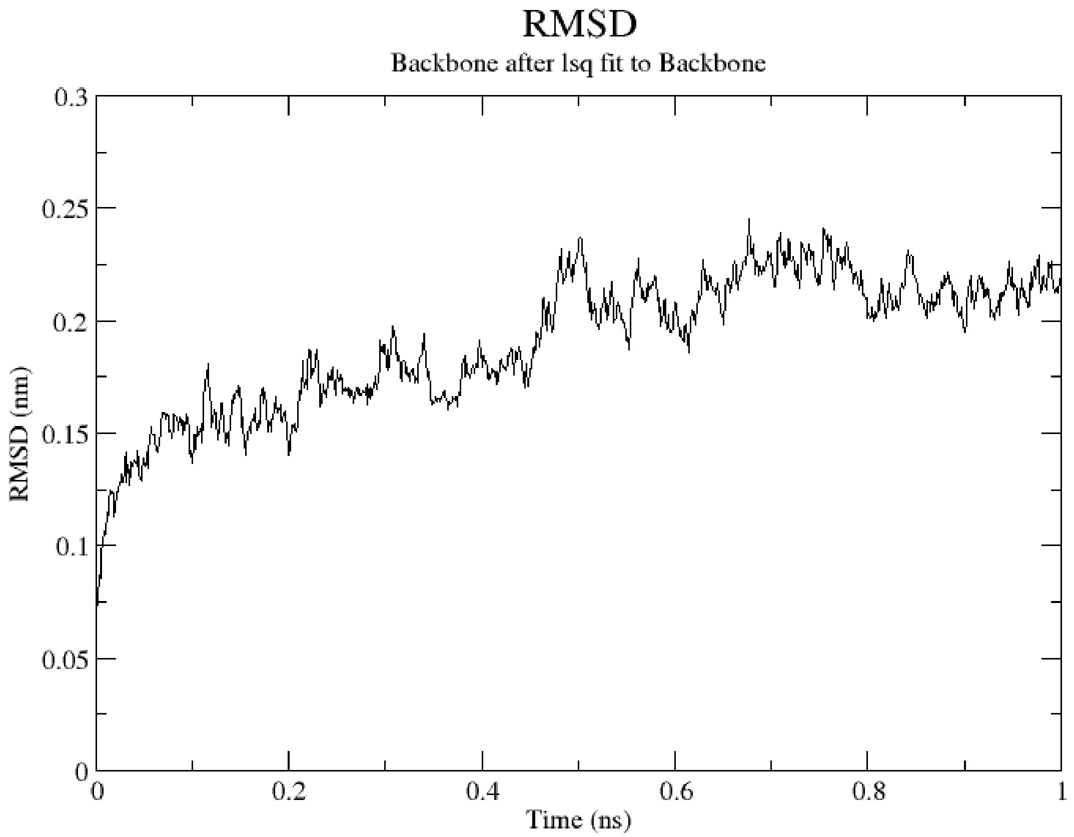

RMSF Plot for 6LU7-TOB

Root mean square fluctuation (RMSF) computes fluctuations (standard deviation) of atomic positions of each amino acids (residues) in the trajectory.

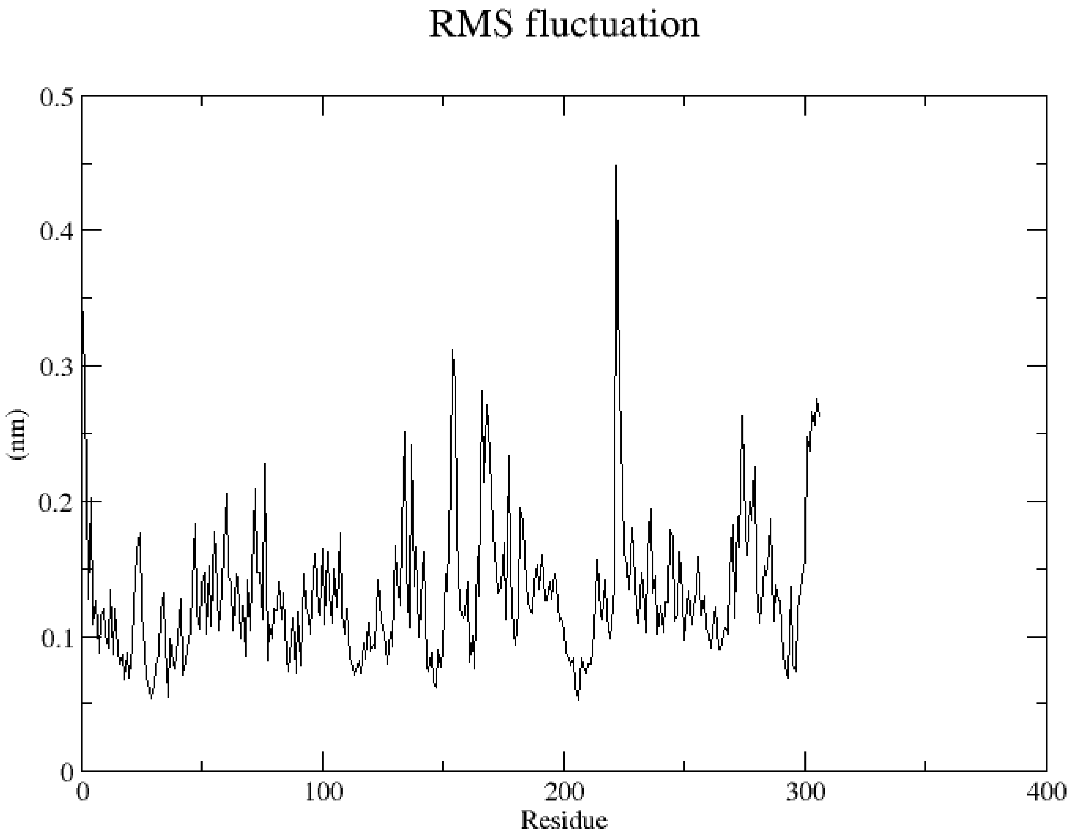

Rg Plot for 6LU7-TOB

Radius of gyration (Rg)computes the radius of gyration (structural compactness) of a molecule and the radii of gyration about the x-, y- and z-axes, as a function of time.

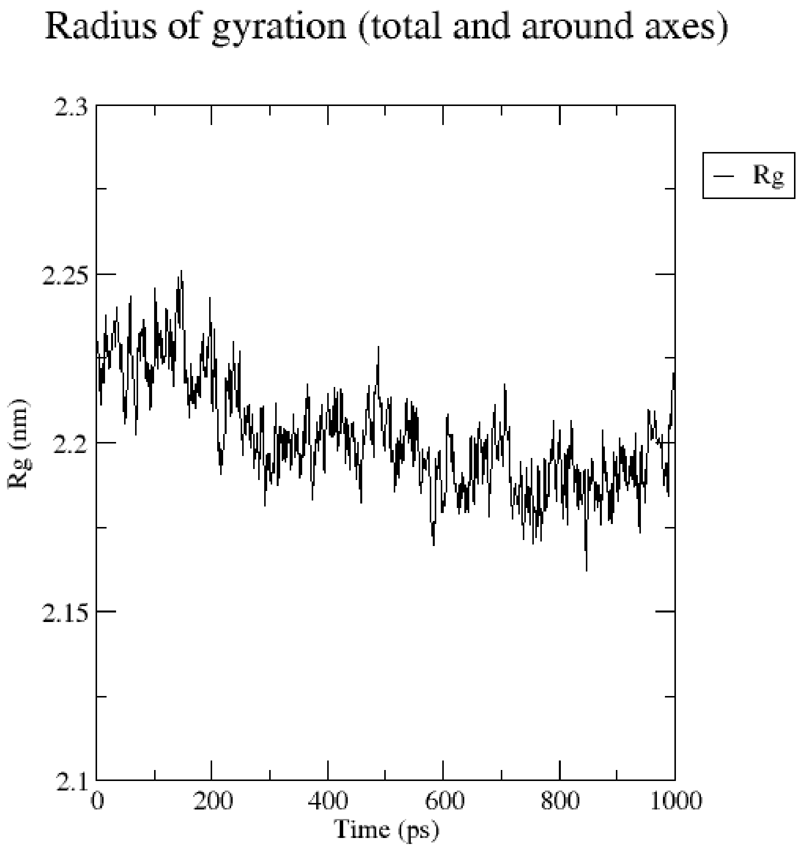

SASA (total) Plot for 6LU7-TOB

Solvent-accessible surface area (SASA) is an approximate surface area of a biomolecule that is accessible to a solvent with respect to simulation time.

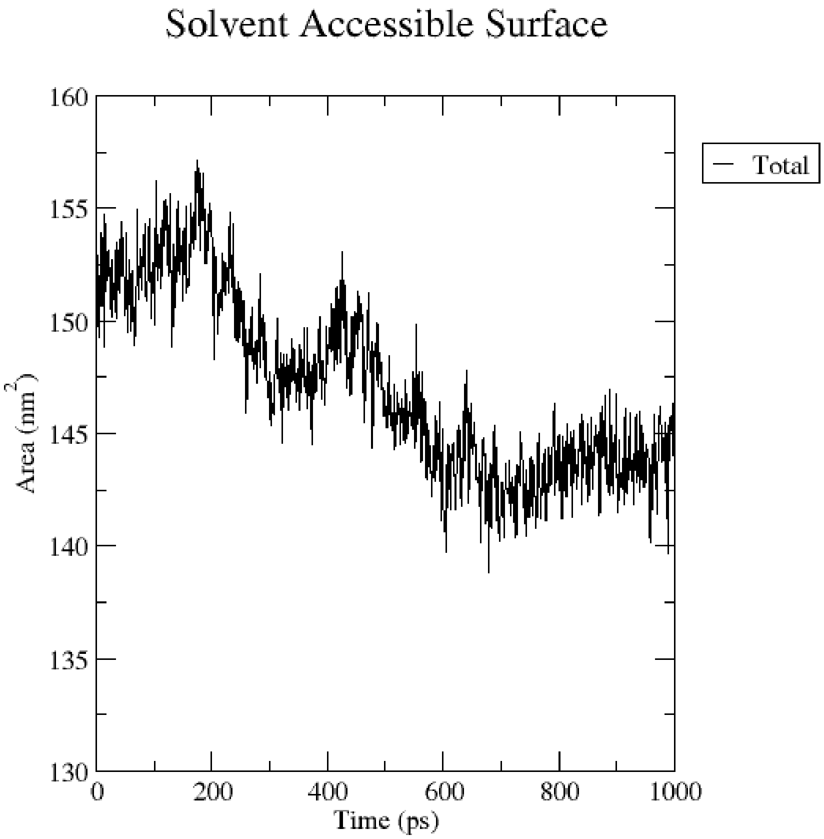

HBONDS Plot for 6LU7-TOB

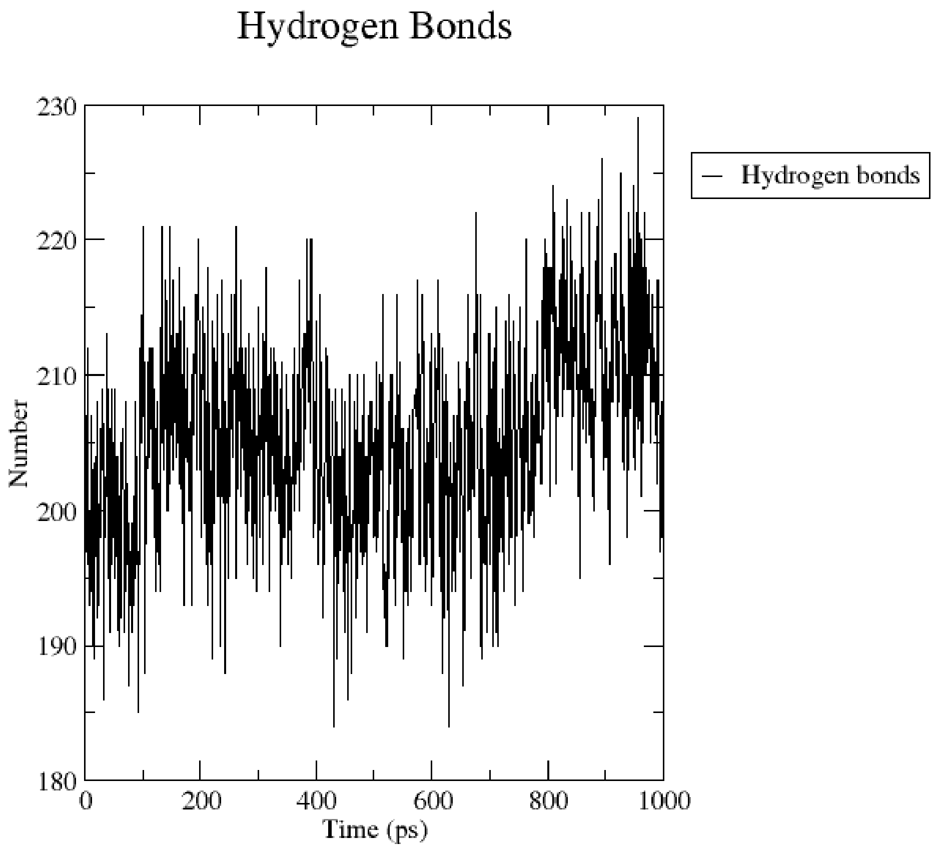

Ligand RMSD Plot for 6LU7-TOB

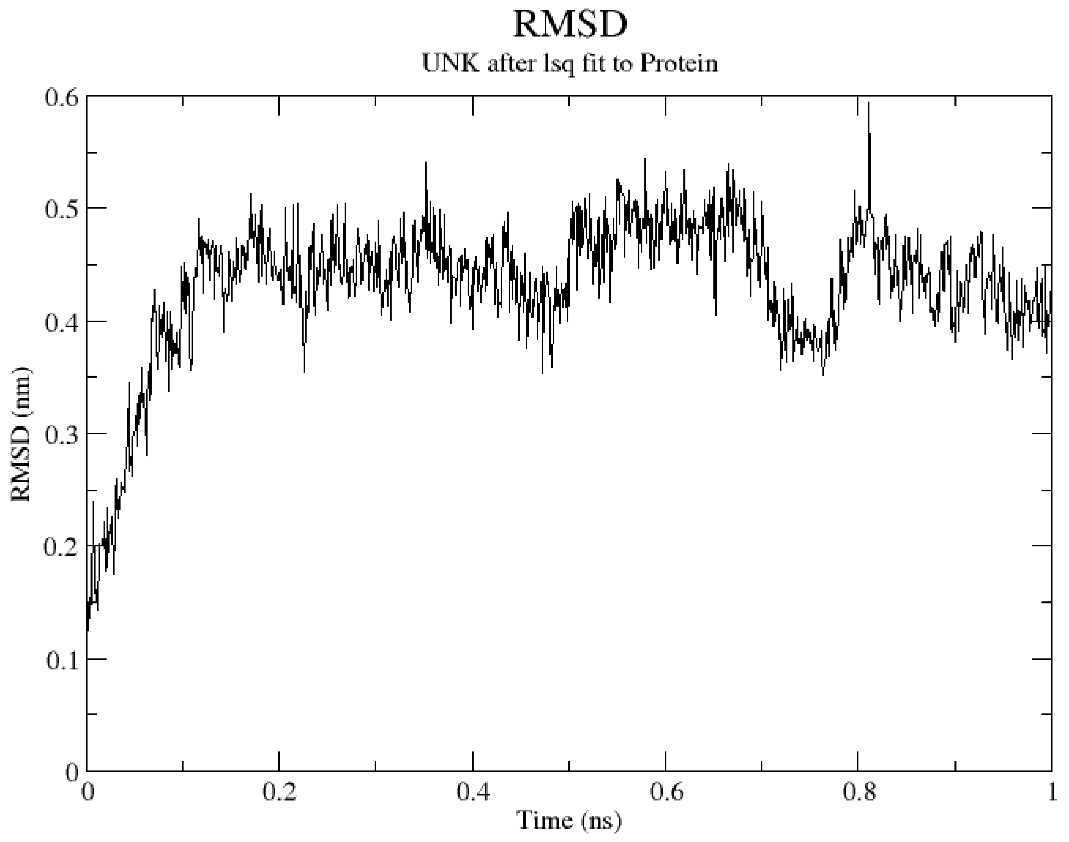

Protein-Ligand HBONDS Plot for 6LU7-TOB

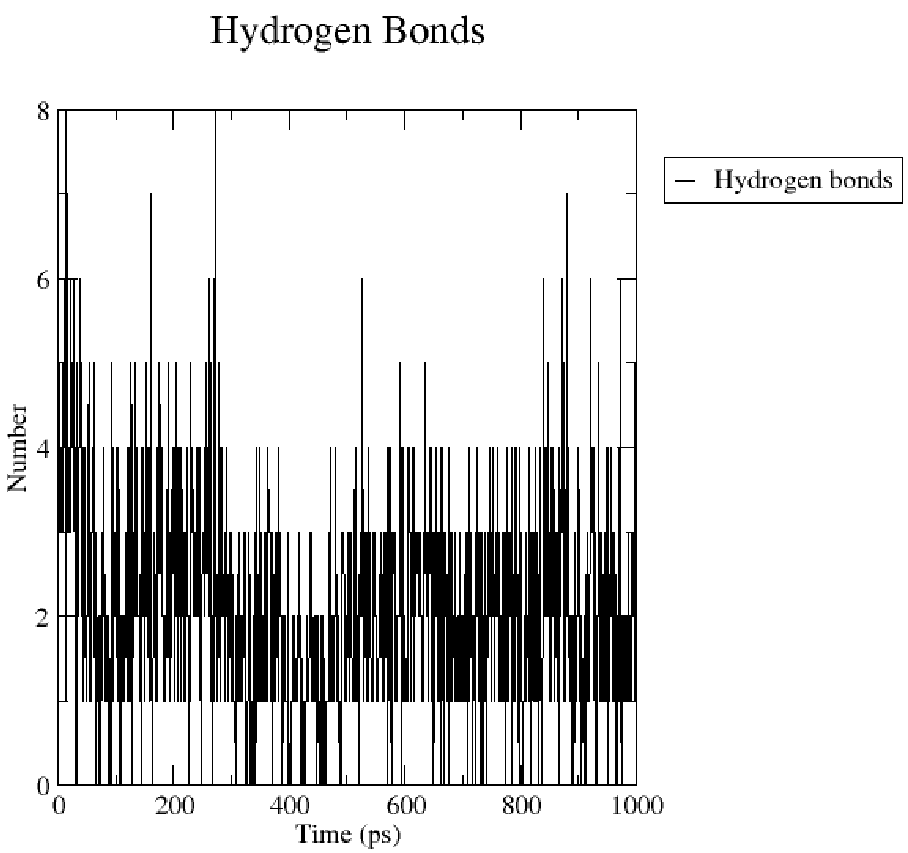
Hydrogen Bonds
Number
0
2
4
6
8
0
200
400
600
800
1000
Time (ps)
Hydrogen bonds
